# Cell-Based Therapy for Fibrosing Interstitial Lung Diseases, Current Status, and Potential Applications of iPSC-Derived Cells

**DOI:** 10.3390/cells13110893

**Published:** 2024-05-22

**Authors:** Yusuke Nakamura, Seiji Niho, Yasuo Shimizu

**Affiliations:** 1 Department of Pulmonary Medicine and Clinical Immunology, Dokkyo Medical University School of Medicine, Mibu 321-0293, Japan; nakamuyu@dokkyomed.ac.jp (Y.N.); siniho@dokkyomed.ac.jp (S.N.); 2 Center of Regenerative Medicine, Dokkyo Medical University Hospital, Mibu 321-0293, Japan; 3 Respiratory Endoscopy Center, Dokkyo Medical University Hospital, Mibu 321-0293, Japan

**Keywords:** fibrosing interstitial lung diseases (FILDs), idiopathic pulmonary fibrosis (IPF), fibrotic hypersensitivity pneumonitis (fHP), induced pluripotent stem cells (iPSCs), alveolar type 2 epithelial cells (AT2s), mesenchymal stem cells (MSCs), stem cells, lung progenitor cells

## Abstract

Fibrosing interstitial lung diseases (FILDs), e.g., due to idiopathic pulmonary fibrosis (IPF), are chronic progressive diseases with a poor prognosis. The management of these diseases is challenging and focuses mainly on the suppression of progression with anti-fibrotic drugs. Therefore, novel FILD treatments are needed. In recent years, cell-based therapy with various stem cells has been investigated for FILD, and the use of mesenchymal stem cells (MSCs) has been widely reported and clinical studies are also ongoing. Induced pluripotent stem cells (iPSCs) have also been reported to have an anti-fibrotic effect in FILD; however, these have not been as well studied as MSCs in terms of the mechanisms and side effects. While MSCs show a potent anti-fibrotic effect, the possibility of quality differences between donors and a stable supply in the case of donor shortage or reduced proliferative capacity after cell passaging needs to be considered. The application of iPSC-derived cells has the potential to overcome these problems and may lead to consistent quality of the cell product and stable product supply. This review provides an overview of iPSCs and FILD, followed by the current status of cell-based therapy for FILD, and then discusses the possibilities and perspectives of FILD therapy with iPSC-derived cells.

## 1. Introduction

Fibrotic interstitial lung disease (FILD) may have various causes and, depending on its etiology and progression, the course of the disease may be fatal. Although some patients may improve with anti-inflammatory treatment such as steroids and immunosuppressive drugs, idiopathic pulmonary fibrosis (IPF), the most common type of FILD, is relatively nonresponsive to such treatments and is often fatal because of its progressive course [1,2]. In cases of severe fibrosis, reversible changes are not expected, and further fibrosis is induced from the fibrotic areas, resulting in the progression of the disease [3]. In such cases, treatment focuses on controlling disease progression, and a radical cure is considered unlikely. Although lung transplantation is sometimes performed in severe treatment-refractory cases, the number of donors is limited, and not all critical patients are eligible to get transplanted [4]. In addition, even if transplantation was performed, survival rates are lower than in healthy or slowly progressing patients [4]. Therefore, there is an urgent and critical need for novel treatment modalities for progressive FILD.

Induced pluripotent stem cells (iPSCs) are artificially induced pluripotent stem cells that can be differentiated into specific cells for use in therapeutic applications [5]. iPSCs themselves have been reported to have anti-inflammatory effects, and studies are evaluating their potential clinical application as a cell-based therapy [6,7,8,9]. iPSCs also have a wide range of potential applications in drug discovery and disease research [10,11]. In recent years, iPSCs derived from various human leukocyte antigens (HLAs) have been prepared and stockpiled from the blood of healthy donors in Japan [12]. Matching these HLAs with recipients is enabling cell therapies with a reduced risk of immune rejection to be considered [12].

In recent years, clinical studies have evaluated the possibility of stem cell-based therapies for FILD. To date, there are no reports of clinical trials on iPSCs for the treatment of FILD; however, preclinical studies in animals have indicated that iPSCs may be effective. Nevertheless, the clinical application of undifferentiated iPSCs still faces a considerable number of challenges.

In this review, we will outline the current state of knowledge about cell-based cell therapy for the treatment of FILD, which has received much attention in recent years, and discuss the potential and future prospects of cell-based therapy with iPSC-derived cells.

### 1.1. An Overview of FILD

The most common cause of FILD is idiopathic pulmonary fibrosis (IPF), but there is a wide range of other causes, including fibrotic hypersensitivity pneumonitis, connective tissue disease-associated interstitial lung disease, pulmonary fibrosis due to sarcoidosis, and chronic drug-induced interstitial lung disease. In each of these diseases, the fibrosis becomes progressive in approximately 20% to 30% of cases, a condition referred to as progressive fibrosing interstitial lung disease (PF-ILD) [13,14]. The prognosis of PF-ILD has been reported to follow a similar pathology to that of fibrosis in the lungs derived from any originating disease, with an annual forced vital capacity (FVC) loss rate of approximately −190 mL/year [14]. IPF is reported to be chronically progressive, with a median survival of 3 to 5 years after diagnosis [1,2], and to have a similar course to progressive FILD [15].

Although the pathogenesis of FILD varies according to the causative disease, the changes that occur as fibrosis develops are reported to be similar. The mechanism of fibrosis is sustained injury to alveolar epithelial cells, and damage to microvascular endothelial cells and interstitium caused by chemical and immunological agents, such as smoking, drugs, autoimmune antibodies, or cells, which result in inflammation and abnormal wound healing in the pulmonary interstitium. Subsequently, fibroblasts, pericytes, epithelial cells, vascular endothelial cells, and fibrocytes are transformed into myofibroblasts, leading to fibrogenesis [3]. Myofibroblasts produce excess extracellular matrix and tissue remodeling occurs, and then the fibrotic lung tissue itself or hypoxia in the fibrotic area leads to the upregulated production of pro-fibrotic cytokines and activation of myofibroblasts, resulting in a loop-like progression of fibrosis [3,16,17,18,19,20,21]. This process involves pro-fibrotic cytokines such as transforming growth factor-β (TGF-β), platelet-derived growth factor (PDGF), and fibroblast growth factor (FGF) [22,23] and causes airway epithelial cell injury, differentiation into myofibroblasts, epithelial–mesenchymal transition, and the tissue deposition of extracellular matrix, which leads to fibrotic formation [24,25]. The fibrosis process also involves abnormalities in lipid metabolism and the disruption of the vascular network [21,25,26].

Although FILD is treated at its source [27], steroid therapy, the most widely used anti-inflammatory treatment in FILD, may lack efficacy [28,29]. Anti-fibrotic drugs such as nintedanib [30] and pirfenidone [31,32] have been used in the hope of suppressing fibrosis progression, but improvement in disease status is often difficult. In cases where patients are refractory to drug therapy, lung transplantation may be considered, but it has many drawbacks, including age restrictions, the limited number of donors and post-transplant immunosuppressive medications, and poor long-term prognosis [33].

As described above, once fibrosis occurs in FILD, it can lead to progressive fibrosis. Given that FILD is often difficult to treat with current drug therapies and treatment options are limited, the development of novel treatment strategies is highly desirable.

### 1.2. An Overview of iPSCs

iPSCs are artificially induced pluripotent stem cells that were initially generated from mouse cells in 2006 and then from human cells in 2007 [34,35]. Pluripotent stem cells are induced by introducing transcriptional factors, including OCT3/4, SOX2, KLF4, and C-MYC, into somatic cells [35]. iPSCs can differentiate into a variety of cell types, and if they are generated from donor cells, pluripotent stem cells can be generated with a lower risk of immune rejection. However, transplanting iPSCs in an undifferentiated state leads to teratoma formation. In addition, this may induce an immune response against them. Therefore, the transplantation of undifferentiated iPSCs is not practical in clinical applications [36,37,38,39]. In response to teratoma issues, some researchers have modified the reprogramming factor C-MYC to L-MYC, which does not promote tumorigenesis [40], and have devised methods to introduce reprogramming factors without integrating them into the genome (e.g., the plasmid method, or adenovirus method that transiently expresses reprogramming genes) [41,42].

The clinical use of iPSC-derived differentiated cells is being explored in various diseases and applications, including age-related macular degeneration [5], Parkinson’s disease [43,44], platelet production [45,46], and heart failure [47,48]. iPSCs have also been used in reproductive medicine, and it has been reported that functional oocytes have been successfully generated from male mice-derived iPSCs [49]. In addition, patient-specific iPSCs have potential as a strategy for drug screening [10,50]. iPSCs have been reported to have potential efficacy in FILD due to their anti-fibrotic properties [6,7,8,9], but as mentioned above, the transplantation of iPSCs themselves is difficult to utilize due to the teratoma formation. However, it is important to investigate the application of iPSC-derived cells for the treatment of FILD.

## 2. Current Status of Cell-Based Therapies for FILD

Over the years, several cells have been reported to act protectively against FILD [6,7,8,9,51,52,53,54]. For example, a study in 2003 by Ortiz et al. showed that when bone marrow-derived mesenchymal stem cells (BM-MSCs) were intravascularly injected into a mouse model of bleomycin (BLM)-induced interstitial lung disease, they migrated to the damaged lungs and transformed into alveolar epithelial type II (AT2)-like cells, leading to a reduction in inflammation and collagen deposition in lung tissue [51]. In addition to BM-MSCs [51,52,53,54,55,56,57,58,59,60,61,62,63,64], cell-based therapy with the following has also been reported to have benefits for pulmonary fibrosis: iPSCs [6,7,8,9], embryonic stem cells (ESCs) [65], resident lung MSCs [66], placenta-derived cell mixture [67,68], amnion MSCs [69], umbilical cord MSCs [70], amniotic fluid stem cells [71], adipose-derived MSCs (AD-MSCs) [72,73,74,75], hematopoietic stem cells (HSCs) [76], AT2 [77,78], lung spheroid cells [79], and prominin-1/CD133(+) epithelial progenitor cells (PEPCEs) [80]. The various cell-based therapies have a similar mechanism of anti-fibrotic action in the lung, with stem cells accumulating at inflammatory sites and reducing pro-fibrotic cytokines, actions that are thought to exert an anti-fibrotic effect. Furthermore, these cell-based therapies have also been reported to be useful in combination with existing anti-fibrotic therapies [64] and thus hold promise as novel therapies that can be added to current treatments.

**Figure 1:**
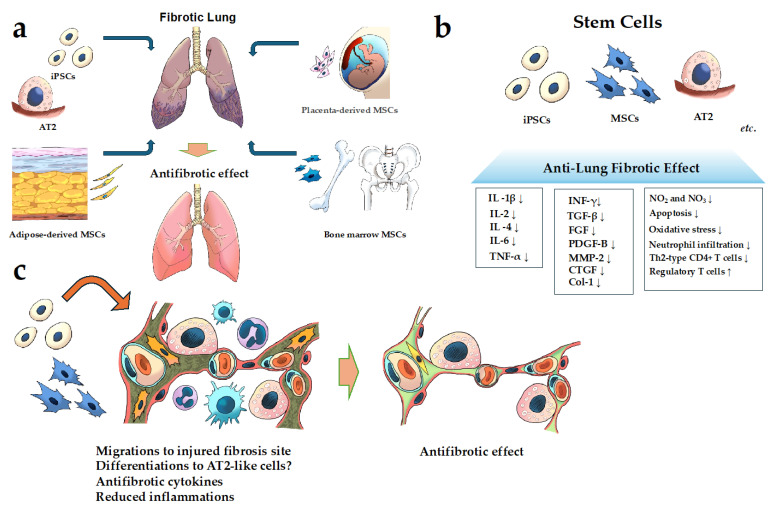
Schematic of cell-based therapy for FILD. (**a**) Examples of the cells used in cell-based therapy in pulmonary fibrosis; (**b**,**c**) the mechanism of the action of the anti-fibrotic effect of stem cells for pulmonary fibrosis. It has been reported that MSCs, iPSCs, and AT2 have anti-fibrotic effects through several mechanisms described in (**b**,**c**). **Abbreviations**: AT2, alveolar epithelial type II; Coll, collagen; CTGF, connective tissue growth factor; IL, interleukin; FGF, fibroblast growth factor; INF-γ, interferon gamma; MMP, matrix metalloproteinase; MSCs, mesenchymal stem cells; PDGF, platelet-derived growth factor; TGF-β, transforming growth factor-beta 1; Th2-type, T helper 2 type; TNF-α, tumor necrosis factor-α. (↑; indicates upregulation or increase. ↓; indicates downregulation or decrease.)

In this section, we will review the current status of cell-based therapies for FILD (summarized in Figure 1 and Table 1).

### 2.1. FILD Treatment with MSCs

MSCs are the most-studied stem cells in cell-based therapy for pulmonary fibrosis. An animal study reported that myelosuppression with busulfan exacerbates fibrosis in a mouse model of BLM-induced interstitial lung disease, suggesting that BM-MSCs may be involved in defense mechanisms against pulmonary fibrosis as a physiological effect [55]. Therefore, BM-MSCs, and other stem cells, may be useful against pulmonary fibrosis.

The anti-fibrotic mechanisms of the action of MSCs are thought to be influenced by factors such as transformation into AT2-like cells (AT2 is involved in alveolar epithelial cell regeneration) [51,58], reduced TGF-β [54,57,61,63,64,69,74,75], reduced FGF [57], reduced PDGF [57], reduced connective tissue growth factor (CTGF) [59,61], the involvement of matrix metalloproteinases (MMPs; with varying increases and decreases, depending on the literature and subtype) [61,69,73,81], reduced inflammatory cytokines (tumor necrosis factor alpha [TNF-α], interleukin [IL]-1α, IL-2, IL-1β, IL-4, and interferon gamma) [55,56,60], increased migration factors (granulocyte colony-stimulating factor [G-CSF] and granulocyte-macrophage colony-stimulating factor [GM-CSF]) [55], the involvement of hepatocyte growth factor (HGF; HGF is responsible for the restoration of alveolar epithelial cells) [53,60], the involvement of stanniocalcin-1 (a mitochondria-related hormone that improves the cell survival) [57], and the involvement of nitric oxide metabolites (the downregulation of NO_2_ and NO_3_, which are thought to exert pro-fibrotic effects) [54]. Further details are provided in Table 1.

Another important issue for stem cell therapy is to find ways to enhance the anti-fibrotic efficacy of cell therapy, and various methods have been investigated with MSCs. One study reported that hypoxic exposure may enhance the anti-fibrotic effect of MSCs by increasing their anti-apoptotic and anti-oxidant factors [59], which can be considered as a potential mechanism for clinical application. Furthermore, culturing MSCs in serum-free media was found to enhance their anti-fibrotic effect, suggesting that the choice of culture medium is also an important aspect to consider [63]. In addition, efforts have been made to enhance the anti-fibrotic effect of MSCs by modifying the molecular biology of the stem cells, and one study investigated the anti-fibrotic effect of MSCs overexpressing miRNAs [62]. In that study, let-7d, a microRNA (miRNA) with anti-fibrotic activity, was overexpressed in MSCs and administered on day 7 after the administration of BLM, which resulted in a faster recovery of weight loss and reduced Col-1 expression, but did not lead to an apparent improvement in fibrosis [62]. Similarly, a study reported an enhanced anti-fibrotic effect of BM-MSCs by the overexpression of HGF [53]. Oncostatin M, which is involved in fibroblast proliferation, has been reported to be elevated in bronchoalveolar lavage fluid from patients with pulmonary fibrosis, and pretreatment with oncostatin M was found to further suppress fibrosis in BLM-induced mouse models [61]. Thus, in addition to the administration of MSCs, exploring methods to enhance their function is also an important issue in cell therapy.

In addition, there is a possibility that MSCs have different effects depending on the age of the donor. In BLM-induced interstitial lung disease mouse models, pulmonary fibrosis showed improvement with AD-MSCs derived from young donor mice (4 months) but not with AD-MSCs derived from aged donor mice (22 months); the authors speculated that the difference in the anti-fibrotic effect of the AD-MSCs was due to the lower fibrotic MMP-2 levels in AD-MSCs from young mice compared with those from old donors [73]. When considering the clinical applications of MSCs, it is necessary to fully assess the origin of the cells and the number of passages.

As described above, many studies have evaluated the use of MSCs in pulmonary fibrosis, and many innovative ways to enhance their efficacy are being investigated.

### 2.2. FILD Treatment with iPSCs

The administration of iPSCs has also been investigated for the treatment of interstitial lung disease [6,7,8,9]. In the model of BLM-induced interstitial lung disease, iPSCs, similar to MSCs, were shown to accumulate in the lung, the site of inflammation, and to have an anti-fibrotic effect [7,8]. Other stem cells, including MSCs and those differentiated from iPSCs into AT2-like cells, also accumulate in the lung at the site of inflammation [8], suggesting that accumulation at the site of inflammation is a cellular property of the stem cells themselves. With respect to the lung, a limited number of reports have described the type of cells that differentiate after the administration of iPSCs. Similarly, relatively few detailed studies have examined the presence of MSCs after cell transplantation, but iPSCs are more pluripotent than MSCs, so they may be more relevant when considering the clinical application of stem cells.

The mechanism of the anti-fibrotic effect of iPSCs is similar to that of other stem cells and is thought to be due to a reduction in inflammatory cytokines such as IL-1, IL-2, IL-10, TNF-α, and monocyte chemotactic protein 1 (MCP1) [7], reduced TGF-β [9], and increased anti-fibrotic chemokine, interferon-gamma-induced protein 10 (IP10) [7]. There have been reports of cytokine changes in the conditioned medium of iPSCs similar to those in iPSCs [7,9], and the factors and pathways by which these changes are induced are the subject of further investigation. Research has found that the TGF-β1/Smad2/3 cascade [9] and the Wnt/β-catenin cascade are involved as signaling pathways in pulmonary fibrosis [6], and the signaling involved in the cell-based therapy of iPSCs for FILD is also becoming increasingly clear. Furthermore, one study showed a significant improvement in survival when iPSCs were administered to BLM-induced mouse models, indicating their possible suitability for clinical applications [7]. The iPSCs used in this study were generated without c-MYC to reduce the tumorigenic risk [83], and the method was aimed at clinical application.

iPSCs have also been reported to be useful in various lung diseases other than FILD. For example, they were found to protect against ventilator-induced lung injury via the nuclear factor-κB pathway [84], against hyperoxia-augmented ventilator-induced lung injury via the Src-dependent signaling pathway [85], against acute lung injury induced by ischemia–reperfusion via the suppression of high-mobility group box-1 [86], an ameliorating effect on hyperoxia-induced lung injury by reducing inflammatory cytokines [87], and to have a protective effect in endotoxin-induced acute lung injury by improving nuclear factor-κB activity and the accumulation of neutrophils [88]. Only animal studies using iPSCs themselves have been reported so far.

The above outlines the use of iPSCs in cell-based therapy for FILD. Although fewer studies have been performed with iPSCs than with MSCs, iPSCs appear to exhibit similar anti-fibrotic effects. However, as mentioned above, clinical application using iPSCs themselves is difficult due to tumor formation.

### 2.3. FILD Treatment with Other Cells

Thus far, we have outlined various studies on cell-based therapy involving MSCs and iPSCs. As previously noted, other stem/progenitor cells have also been reported to have anti-fibrotic properties. For example, AT2 cells were found to act as progenitor cells in the adult lung and to contribute to alveolar repair in the event of lung injury [89] and the intratracheal administration of AT2 in BLM-induced interstitial lung disease mouse models localized to the lung, the site of injury, and suppressed lung fibrosis [77,78].

HSCs have also been studied in the treatment of FILD [76], and the administration of HSCs overexpressing keratinocyte growth factor to BLM-induced pulmonary fibrosis mouse models suppressed fibrosis by reducing TNF-α, CCL-2, and CCL-9 and increasing surfactant protein C-positive cells (AT2 cells) [76].

Cell therapy has also been performed with PEPCs and has shown anti-fibrotic activity against BLM-induced pulmonary fibrosis in mouse models. PEPCs, which are thought to be derived from the bone marrow, localize to the lung, and are thought to have anti-fibrotic effects, thereby differentiating into AT2 and upregulating inducible nitric oxide synthase [80]. Other reports suggest that lung spheroids (formed by MSC, alveolar epithelial cells type I [AT1], AT2, and club cells) also suppress fibrosis [79].

Some studies have reported on the use of differentiated cells derived from iPSCs [8] and ESCs [65] in models of interstitial lung disease, where the intratracheal administration of differentiated AT1 and AT2 derived from iPSCs in BLM-induced mouse models resulted in the suppression of fibrosis [8]. These results showed a significant improvement in the differentiated cell-treated group compared with undifferentiated iPSCs, suggesting the potential use of iPSC-derived differentiated cells for cell therapy [8]. In yet another report, MSCs differentiated from ESCs were studied in pulmonary fibrosis and showed the suppression of fibrosis [82]. The MSCs used in this study were developed by a pharmaceutical company and are already being tested in clinical research on COVID-19 [90].

As described above, various types of stem/progenitor cells other than MSCs and iPSCs are being studied as cell-based therapies for the treatment of FILD. Despite the fact that many types of stem cells have been reported to have protective effects against FILD, to our knowledge no studies have compared the anti-fibrotic effects of each stem cell type on a large scale. Therefore, when considering clinical applications, the selection of stem cells to be used may also be relevant.

### 2.4. Clinical Trials

Clinical research has been performed in IPF, the most common type of FILD, with AD-MSCs stromal vascular fraction [91,92], placental MSCs [93], AT2 [89], and BM-MSCs [94,95,96]. Table 2 summarizes the clinical trials performed in recent years in FILD (or IPF). 

Between 2013 and 2018, phase I clinical trials were performed primarily to confirm safety. The studies ranged in duration from 4 weeks to 2 years and evaluated adverse events, mortality, progression-free survival, lung function, and exercise capacity. None of the studies showed any apparent adverse events, and the 2-year median survival and disease progression were comparable to the epidemiologic data [92].

Subsequently, a randomized phase I/IIA clinical trial with allogeneic human BM-MSCs in IPF patients was reported in 2020, the first of its kind to compare BM-MSC treatment with placebo in IPF patients [96]. On the basis of previous reports, this study used high-dose allogeneic BM-MSCs (2 × 10^8^ cells) [95], and it assessed safety, tolerability, and efficacy as the endpoints [96]. The study found no substantial differences in significant adverse events or mortality between the two groups and showed efficacy in the BM-MSC-treated group in the 6 min walk distance (at 13 weeks), diffusing capacity of the lung for carbon monoxide (at 26 weeks), and FVC (at 39 weeks), and FVC at 52 weeks was higher than the baseline FVC [96]. Considering that the goal of FILD treatment with compounds such as pirfenidone and nintedanib is to curb the loss in FVC, the finding that cell therapy with MSCs also resulted in an increase in FVC was a decisive step forward from previous therapies. This study concluded that therapy with high doses of allogeneic MSCs was a safe and promising method to reduce disease progression [96].

As described above, phase II clinical trials have investigated cell therapies for IPF, and although they have collected comparative data only in a small number of cases, they have demonstrated the usefulness of BM-MSCs. Despite the limited number of studies, BM-MSCs are currently considered to be an effective candidate for cell-based therapies.

## 3. Points of Concern with Recent Cell-Based Therapy

As mentioned above, MSCs can be derived from a variety of organs, and AD-MSCs in particular have a high proliferative capacity and well-established culture techniques. However, the number of proliferations is limited compared to iPSCs. In other words, there is a potential risk of supply shortages. In addition, the efficacy of AD-MSCs may vary depending on the age of the donor from which they are derived [73]. Therefore, it is not simply a question of whether any AD-MSCs are acceptable. This suggests that it is difficult to maintain the homogeneity of quality.

BM-MSCs, which have been used in many clinical trials for FILD to date, are widely used and there are also concerns about their stable supply due to the limited number of donors with this invasive procedure. Placental MSCs and AT2 have similar problems, making it difficult to obtain enough cells for clinical application.

Because of these problems, iPSCs with high self-renewal potential may be a useful option as a cell source for FILD cell-based therapy. The use of iPSCs may solve the problems of consistent quality and stable supply associated with the use of MSCs. Previous reports about cell-based therapy have also highlighted the importance of product quality [97].

## 4. The Problems of Using iPSCs Themselves to Treat FILD

Many papers have reported that stem cells, including iPSCs, exhibit anti-fibrotic effects in the lung, and some MSCs have been found to be useful in clinical applications. Although the usefulness of iPSCs in terms of their anti-fibrotic effects has been reported, clinical applications have not yet been developed. As described above, there are several challenges to the clinical application of iPSCs themselves, particularly concerns about tumorigenesis [38] and the difficulty of accurately assessing differentiation after the administration of iPSCs. Although there is evidence that iPSCs transplanted for FILD accumulate in the lung, the site of inflammation [8], they are systemically disseminated when administered intravascularly. Because pluripotent stem cells such as iPSCs can be induced to differentiate into various cell types depending on the stimuli of the environment in which they are seeded, the administration of undifferentiated iPSCs is virtually infeasible as a clinical application unless their differentiation is assessed after administration. For example, if iPSCs inadvertently differentiate into hematopoietic stem cells, there may be a risk of future graft-versus-host disease. For this reason, clinical applications that use iPSCs themselves will be difficult to develop unless the cells demonstrate greater efficacy than MSCs or other stem cells with safety. 

Although there is a paucity of the literature comparing the anti-fibrotic effects of iPSCs and differentiated cells, some studies have compared the anti-fibrotic effects of iPSCs and iPSC-derived AT2-like cells and found stronger anti-fibrotic effects in the latter [8], making the use of differentiated cells derived from iPSCs an option to be considered.

## 5. Possibility for iPSC-Derived Cell-Based FILD Therapy

Despite the problems associated with the clinical application of iPSCs themselves, these derived cells have advantages in cell-based therapy for the treatment of FILD. As mentioned previously, the stockpiling of various HLAs has begun in Japan, and the use of matching HLAs has the potential to complement not only the anti-inflammatory but also the functional effect of stem cell-based therapy [12]. MSCs and AT2, which have shown anti-fibrotic effects, have already been reported in the methods to differentiate from iPSCs [98,99]. Therefore, it is now conceivable to target drug development with these iPSC-derived cells with matched HLA.

As mentioned above, the drug supply for current cell-based therapies is dependent on the number of donors, which is a potential risk for stable supply. The proliferative capacity of iPSCs is not expected to decrease with culture passaging, unlike MSCs, which have been more extensively studied for cell-based therapy in pulmonary fibrosis. The use of iPSC-derived cells may also reduce the risk of quality variation between donors for the derivation of other stem cells (e.g., MSCs or AT2) and allow for a more stable product supply. In addition, both the selection of the source cells that show the strongest anti-fibrotic effect in FILD and the selection of the differentiated cells in the generation from iPSCs can be considered. This, in turn, could lead to the creation of cell products with greater anti-fibrotic activity than existing MSCs and AT2 cell therapies.

In addition, the clinical applications of iPSC-derived cells have already been underway [5], and iPSC-derived cells will not be an ethical or technical limitation. The use of differentiated cells derived from iPSCs is expected to reduce the risk of tumor formation, which has been a concern with iPSCs.

**Figure 2:**
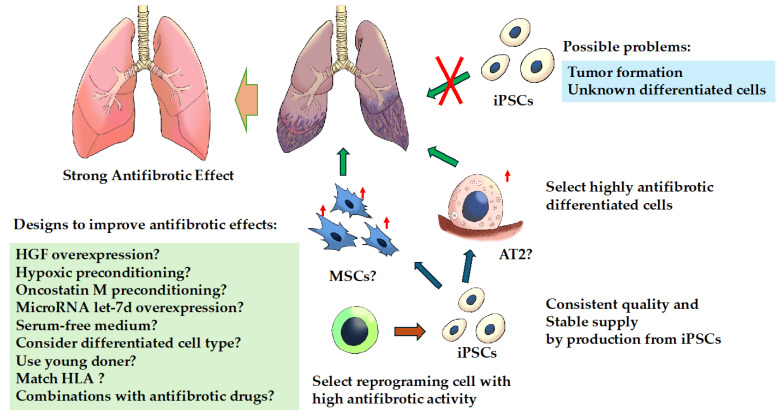
Treatment strategies for fibrosing interstitial lung diseases with induced pluripotent stem cells. Cell-based therapy with induced pluripotent stem cells (iPSCs) per se is likely to be difficult for several reasons. There is a possibility that effective cell-based therapy can be achieved by inducing iPSCs from cells with efficient anti-fibrotic activity and differentiating them into cells with high anti-fibrotic activity because iPSCs may retain the characteristics of the parent cells from which they were generated. **Abbreviations**: AT2, alveolar epithelial type II; HGF, hepatocyte growth factor; iPSCs, induced pluripotent stem cells; MSCs, mesenchymal stem cells. ↑: indicates enhanced.

In light of the above, although there are difficulties in applying iPSCs themselves in cell therapy, if cells with high anti-fibrotic activity can be generated by further studying the origin of iPSCs and differentiated cells, such cells may be found to have an anti-fibrotic effect that is superior to that of the existing cell therapies (Figure 2).

## 6. Proposal for iPSC-Derived Cell Type for FILD Treatment

The properties of iPSCs may differ depending on their origin, and in particular, low-passage-generated iPSCs have been reported to exhibit DNA methylation features similar to those of the somatic cells from which they were derived [100]. For example, such changes may facilitate differentiation into the derived cells and have indeed been used in animal experiments [101]. This means that when iPSCs are reprogrammed, the epigenetic changes are similar to the cells from which they are derived, and they are more likely to differentiate into their original cells. It is therefore desirable, in terms of differentiation efficiency, that the post-differentiated cells and the cells to be reprogrammed are identical.

MSCs have been reported to differ in gene expression depending on the tissue or organ from which they are harvested [102] in a study comparing umbilical cord blood-derived neonatal unrestricted somatic stem cells (USSCs), BM-MSCs, and AD-MSCs. USSCs showed gene expression related to neurogenesis, BM-MSCs showed a tendency to differentiate into mesoderm and ectoderm, and AD-MSCs were highly enriched in immune-related genes [102]. Because of these differences, the reprogramming cell source should also be taken into account when MSCs are to be considered as differentiated cells. If immunomodulatory effects are expected, it may be possible to produce cell products with high anti-fibrotic effects by using AD-MSCs as reprogramming cells, then generating iPSCs and differentiating them into AD-MSC-like cells. Other studies have reported that AD-MSCs secrete more HGF (responsible for the restoration of alveolar epithelial cells) and VEGF in the low serum culture system, and these may have a higher regenerative capacity and immunoregulatory function than BM-MSCs [103,104]. While many previous reports have used BM-MSCs, AD-MSCs may be more suitable. (↑; indicates enhanced.)

There are reports that both AD-MSCs and BM-MSCs are similar in terms of phenotype and differentiation [105], and it would be difficult to classify the molecular biology of the two. For example, it has been reported that the cytokine production and the gene expression involved in initiation vary with passaging. This further complicates the distinction between the two MScs. However, one report mentioned that the production of IL-6 by BM-MSCs is low in early passages, whereas AD-MSCs produce high levels, which is a possibility to be specific. It may be a result of being influenced by the location of the adipose tissue harvested, and further studies are needed to differentiate them. Despite these differences, both AD-MSCs and BM-MSCs are effective iPSC-derived cell options because they exert anti-fibrotic effects through similar mechanisms. AT2 has also been considered as a target for the differentiation of iPSCs. Although both MSCs and AT2 have been studied in clinical trials [92,95,96], there are no comparative studies between them, including basic studies, and future studies are needed.

At present, MSCs are most likely to be considered, given the abundance of previous reports and clinical trials on FILDs, and AD-MSCs, which have been reported to secrete high levels of HGF, are recommended as differentiated cells.

## 7. Limitations for iPSC-Derived Cell

The exact mechanism by which the differentiated cells exert their anti-inflammatory effects is not fully understood. Many studies have shown that these cells accumulate at the sites of inflammation and exert their anti-inflammatory effects, but there are some points to consider. 

The first concern is the difficulty in assessing cell differentiation after the cell products have been administered. MSCs have “multipotency” to differentiate into multiple cell types, which is different from the “pluripotency” characteristic of iPSCs. However, for example, several reports have shown that administrated BM-MSCs were differentiated into AT2-like cells [51,58]. As these results have been scarce in recent years, the frequency of differentiation into AT2-like cells may not be high or strongly involved in anti-fibrotic effects. However, the fact that administered cells differentiate is certain, and assessing the distribution of all administered cells is difficult.

Another concern is that it is not fully understood whether the cells are eliminated or whether they differentiate and survive after administration at the site of inflammation. When transplanting human-derived MSCs into mice, engraftment varies depending on the immunodeficient condition of the mouse, thus rejection must be considered [52]. MSCs generally do not express MHC class II and therefore have low antigenicity and are not eliminated in the short term (48 h of INF-γ exposure upregulated MHC class II in MSC, but no lymphocyte alloreactivity was seen) [106]. On the other hand, long-term course and MSC-derived cells may be eliminated in the case of MHC-mismatched cell transplantation [107,108]. However, it does not suggest that the anti-fibrotic effect is lost with MSC rejection after a long period of administration. Human BM-MSCs showed anti-fibrotic effects even when transplanted into normal immunocompetent C57BL/6J BLM mouse models [57]. The MSCs are unlikely to be viable in the long term, and if they are thought to act in the short term then they may be eliminated. In other words, it is thought that an anti-inflammatory effect could be expected in the lung if it acts even for short periods during high levels of inflammation. As another example, bone marrow-derived “allogeneic” multipotent adult progenitor cells (invimestrocel) have been reported to potentially improve 180-day survival in acute respiratory distress syndrome (ARDS) [109]. Whether the transplanted cells engraft is an important question, but the initial anti-inflammatory effects of stem/progenitor cells may lead to a better long-term outcome, even if they are rejected. Although the degree of antigenicity of this cell is unknown and whether it is eliminated or engrafted cannot be determined, an assessment of the health risk of long-term engraftment is also necessary.

## 8. Conclusions

At present, the clinical use of undifferentiated iPSCs is likely to be difficult for a number of reasons that need to be addressed before iPSCs themselves can be introduced as therapeutic agents in cell-based therapy for FILD. However, regenerative medicine products generated from differentiated cells derived from iPSCs appear to hold promise as innovative cell-based therapies in the future.

## Figures and Tables

**Table 1:** Preclinical studies on cell-based therapy for fibrosing interstitial lung diseases with stem cells. Summarizes the progress to date in cell-based therapy for FILD.

Preclinical Studies								
**Bone Marrow MSCs**								
	**Year**	**Model**	**Source**	**Effect**	**Effect 2**	**Outcome**	**Ref** **.**	
	2003	BLM mouse	Mouse BM MSCs	Differentiate into AT2-like cells	Decrease matrix metalloproteinases	Reduced inflammation and col I deposition	Proc Natl Acad Sci U S A. 2003;100(14):8407-11.	[51]
	2005	Myelo-suppressed BLM mouse (i.e., has increased susceptibility)	Mouse BM MSCs	Localized in the lung with AT2 or other cell phenotypes of the lung	G-CSF and GM-CSF ↑; IL-2, -1β, -4, and INF-γ ↓	Reduced inflammation and fibrosis	Am J Respir Cell Mol Biol. 2005;33(2):145-52.	[55]
	2007	BLM mouse	Mouse BM MSCs	IL-1 receptor antagonist production	Protect from injury by blocking TNF-α (Mφ) and IL-1α (T cells)	Decrease in TNF-alpha and IL-1 alpha	Proc Natl Acad Sci U S A. 2007;104(26):11002-7.	[56]
※	2008	BLM mouse (NOD/SCID, NOD/SCID/β2M)	Human BMDCs			BMDCs were more migrated in NOD/SCID/β2M mouse	Am J Physiol Lung Cell Mol Physiol.2008;295(2):L285-92.	[52]
	2010	BLM rat	Rat BM MSCs	NO_2_ and NO_3_ ↓ in lung	IL-1β, VEGF, IL-6, TNF-α, and TGF-β ↓ in lung	Reduced inflammation and fibrosis	Respir Res. 2010;11(1):16.	[54]
※	2013	BLM rat and human UIP lung	Human BM MSCs (HGF overexpression)	MSC (HGF+, marrow-derived) presented in lung	Wet lung volume and Ashcroft score ↓	Reduced inflammation and fibrosis	PLoS One. 2013;8(6):e65453.	[53]
	2014	BLM mouse	Human BM MSCs (UE6E7T-2) and the inhalation of STC1	STC1 secretion from MSCs by TGF-β	Col synthesis, oxidative stress, TGF-β1, FGF2, and PDGF-B ↓	Reduced inflammation and fibrosis	Mol Ther. 2015;23(3):549-60.	[57]
	2015	BLM rat	Mouse BM MSCs	Differentiate into AT2-like cells (surfactant protein C ↑)	Increase antioxidative capability	Reduced fibrosis	Mol Med Rep. 2015;11(3):1685-92.	[58]
※	2015	BLM mouse; MSC and fibroblast coculture	Mouse BM MSCs (hypoxic preconditioning)	HIF-1α, HGF, VEGF, and HO-1 ↑ by hypoxic preconditioning of MSC	HGF, CTGF, and col I amount ↓ in the lung (dominant in preconditioning)	Reduced inflammation and fibrosis	Stem Cell Res Ther. 2015;6(1):97.	[59]
※	2016	BLM mouse	Mouse BM MSCs (HGF overexpression) and MSC CM (probably HGF)	MSC CM: apoptosis inhibition	MSC: IL-1β ↓ and HGF ↑; MSC ΔHGF: none	Reduced inflammation and fibrosis	Stem Cells Transl Med. 2016;5(10):1307-1318.	[60]
※	2017	BLM mouse	Mouse BM MSCs (Oncostatin M preconditioning)	HGF ↑ by oncostatin M preconditioning of MSC	Col III, CTGF, MMP9, TIMP1, and TGF-β1 ↓ in the lung (dominant in preconditioning)	Reduced inflammation and fibrosis	Stem Cells Transl Med. 2017;6(3):1006-1017.	[61]
※	2017	BLM mouse	Human BM MSCs (let-7d [antifibrotic] or miR-154 [profibrotic] overexpression)	Recover quicker from weight loss, col I, and CD45+cells ↓ (let-7d)	Survival rate ↓ (miR-154)	The possibility of miRNA-modified MSC	Am J Physiol Lung Cell Mol Physiol.2017;313(1):L92-L103.	[62]
※	2021	BLM rat	Rat BM MSCs ± nintedanib	Homing to injured lung	TGF-β/SMAD3 signaling, TNF-α, and IL-6 ↓ (dominant in BM MSCs + nintedanib)	Reduced inflammation and fibrosis	Inflammation. 2020;43(1):123-134.	[64]
※	2021	BLM mouse	Human BM MSCs with serum-free media	Lung engraftment and Treg ↑	TGF-β1 and IL-6 ↓	Reduced inflammation and fibrosis	Stem Cell Res Ther. 2021;12(1):506.	[63]
**Adipose tissue MSCs**								
	**Year**	**Model**	**Source**	**Effect**	**Effect 2**	**Outcome**	**Ref**	
	2014	BLM mouse	Human AD MSCs	Apoptosis ↓ and TGF-β1 ↓	The hyperplasia of Club cells, infiltration of the perialveolar ducts by inflammatory cells, septal thickening, enlarged alveoli, Ashcroft score, and hydroxyproline ↓	Reduced inflammation and fibrosis	Exp Lung Res. 2014;40(3):117-25.	[74]
※	2015	BLM mouse	Mouse AD MSCs (young or adult donor)	Fibrosis, MMP-2 activity, oxidative stress, and the markers of apoptosis ↓ (dominant in young)	MMP-2, IGF receptor, and protein kinase B (AKT) ↑ in young donor	Reduced inflammation and fibrosis	Transl Res. 2015;166(6):554-67.	[73]
	2017	BLM mouse	Mouse AdSC	TNF-α and IL-12 ↓ and apoptosis ↑ (Mφ)	Th2-type CD4+ T cells ↓ and regulatory T cells ↑	Reduced inflammation and fibrosis	Sci Rep. 2017;7(1):14608.	[72]
	2023	BLM mouse	Mouse AD-MSCs and the CM of AD-MSCs	Ashcroft score, hydroxyproline ↓; fibroblast proliferation and migration ↑ (in vitro MSC and CM)	TGF-β, αSMA, and Col I ↓ (in vitro, CM); CM is a key?	Reduced inflammation and fibrosis	Sci Rep. 2023;13(1):13183.	[75]
**Placental MSCs**								
	**Year**	**Model**	**Source**	**Effect**	**Effect 2**	**Outcome**	**Ref**	
	2009	BLM mouse	Human placenta-derived cells, 50% mesenchymal cells (AMSCs + CMSCs), and 50% epithelial cells (hAECs)	Homing to injured lung	Neutrophil infiltration and the severity of lung fibrosis ↓	Reduced inflammation and fibrosis	Cell Transplant. 2009;18(4):405-22.	[67]
	2009	BLM mouse	Human UC MSC	TGF-β, IL-10, INF-γ, and col Ia ↓	MMP2 ↑	Reduced inflammation and fibrosis	Am J Pathol. 2009;175(1):303-13.	[70]
	2013	BLM mouse	Human AM MSC, BM MSCs, and hAECs	IL-1 (AM-MSC), IL-6 (AM-MSC, BM-MSC, hAEC), and TNF-a (AM-MSC) ↓	MMP-9 (AM-MSC) ↑ and TGF-β (AM MSC, BM MSC, and hAEC) ↓	Reduced inflammation and fibrosis (AM MSC)	PLoS One. 2013;8(8):e69299.	[69]
	2013	BLM mouse	Mouse AFSC	CCL2 ↓; MMP2 (transiently) ↑	AFSC migration to fibrosis lesion and MMP-2 was associated with the cleavage of CCL2	Reduced inflammation and fibrosis	PLoS One. 2013;8(8):e71679.	[71]
	2017	BLM mouse (MyD88-deficient)	Human PL MSC	ΔmyD88 in BLM mouse indicates reduced fibrosis (MyD88 might be related to fibrosis)	Hydroxyproline, MyD88, and TGF-β signaling ↓	Reduced inflammation and fibrosis	Mol Immunol. 2017:90:11-21.	[68]
	2023	BLM rat	Human UC MSC vs. human AD MSC	Lung function and blood oxygen saturation ↑; cell number and myofibroblast activation ↓ (BALF) in UC-MSC	MMP-9 and Toll-like receptor-4 (alveolar regeneration) ↑ in UC MSC	Reduced inflammation and fibrosis	Int J Mol Sci. 2023;24(8):6948.	[81]
**iPS**								
	**Year**	**Model**	**Source**	**Effect**	**Effect 2**	**Outcome**	**Ref**	
	2013	BLM mouse	Mouse iPSCs and iPSC CM	Ashcroft score, col I, hydroxyprolines, and neutrophil ↓	IL-1, IL-2, IL-10, TNF-α, and MCP1 ↓; IP-10 ↑	Reduced inflammation and fibrosis, and survival ↑	Shock. 2013;39(3):261-70.	[7]
	2014	BLM mouse	Mouse iPSC and iPSC-derived AT1- and AT2-like cells	iPSCs and differentiated cells migrate to the injured region	IL-6 (BALF), TNF-α (BALF), and hydroxyproline ↓ (iPSC-derived AT2 > iPSC)	Reduced inflammation and fibrosis	Stem Cells Transl Med. 2014;3(6):675-85.	[8]
	2016	BLM mouse	Mouse iPSCs	MMP-2, IL-1β, IL-6, iNOS, NO, and COX2 ↓	TGF-β1/Smad2/3 signaling and epithelial to mesenchymal transition ↓	Reduced inflammation and fibrosis	Front Pharmacol. 2016:7:430.	[9]
	2023	BLM mouse	Mouse iPSCs	Hydroxyproline ↓	Wnt, β-Catenin, and LEF ↑; DKK1 ↓ in the lung	Reduced inflammation and fibrosis	Stem Cell Res Ther. 2023;14(1):343.	[6]
**AT2**								
	**Year**	**Model**	**Source**	**Effect**	**Effect 2**	**Outcome**	**Ref**	
	2007	BLM rat	Rat AT2	Homing to injured lung	Lung hydroxyproline ↓	Reduced inflammation and fibrosis	Am J Respir Crit Care Med. 2007;176(12):1261-8.	[77]
	2014	BLM rat	Rat AT2	Homing to injured lung	The recovery of surfactant proteins	Reduced inflammation and fibrosis	J Heart Lung Transplant. 2014;33(7):758-65.	[78]
**The others**								
	**Year**	**Model**	**Source**	**Effect**	**Effect 2**	**Outcome**	**Ref**	
	2009	Lethally irradiated BLM mouse	Lineage negative HSCs + KGF overexpression	Proliferative AT2 (surfactant protein C ↑)	TNF-α, CCL-2, and CCL-9 ↓	Reduced inflammation and fibrosis	PLoS One. 2009;4(11):e8013.	[76]
	2009	BLM mouse	PEPCs (epithelial progenitor cells)	PEPCs were of bone marrow origin; differentiated into AT2-like (SP-C+); inducible nitric oxide synthase ↑	IL-4, IL-6, IL-13, and TNF-α, MCP1 ↓ (day 7); TGF-β, fibronectin, and col I↓ (day 21)	Reduced inflammation and fibrosis	Am J Respir Crit Care Med. 2009;179(10):939-49.	[80]
	2011	BLM mouse	Mouse LuMSCs	BLM depletes the endogenous LuMSCs that regulate effector T-cell proliferation	Ashcroft score and survival ↑	Reduced inflammation and fibrosis	Stem Cells. 2011;29(4):725-35.	[66]
	2012	BLM mouse	Human ESCs (H7)-derived AT1, AT2, and Clara-like cell mixture	Col Ia, TGF-β1, FGF, and VEGF-A ↓	Col amount ↓	The possibility of using ESCs to treat fibrosis	PLoS One. 2012;7(3):e33165.	[65]
	2020	BLM rat	rat LCS (MSC, AT1, AT2, and club cell)	Homing to injured lung, Ashcroft score ↓	Apoptosis ↓ and angiogenesis ↑; the protection of AT1 and AT2 cells	Reduced inflammation and fibrosis	Stem Cells Transl Med. 2020;9(7):786-798.	[79]
	2023	BLM mouse	DW MSCs (derived from human ESCs)	Ashcroft score and col amount ↓; Acta2, col Ia, CTGF, TGF-β, and IL-1b ↓,	Anti-apoptotic effects by transferring their mitochondria	Reduced inflammation and fibrosis	Immune Netw. 2023;23(6):e45.	[82]

**Abbreviations:** AD-MSCs, adipose MSCs; AdSC, adipose-derived stem cell; AFSC, amniotic fluid stem cell; αSMA, alpha smooth muscle actin; AM MSC, amnion mesenchymal stem cells; AT2, alveolar epithelial type II; BALF, bronchoalveolar lavage fluid; β2M, β2 microglobulin; BLM, bleomycin; BMDCs, bone marrow-derived cells; BM MSC, bone marrow mesenchymal stem cells; Col, collagen; CM, conditioned medium; CMSC, chorionic mesenchymal stromal cells; COX2, cyclooxygenase-2; CTGF, connective tissue growth factor; DW, Daewoong Pharmaceutical; DKK1, dickkopf-1; ESCs, embryonic stem cells; FGF, fibroblast growth factor; G-CSF, granulocyte colony stimulating factor; GM-CSF granulocyte-macrophage colony-stimulating factor; hAECs, human amniotic epithelial cells; HIF-1α, hypoxia-inducible factor-1α; HGF, hepatocyte growth factor; HO-1, anti-heme oxygenase 1; HSCs, hematopoietic stem cells; IGF, insulin-like growth factor; IL, interleukin; IMMP, matrix metalloproteinase; iNOS, inducible nitric oxide synthase; iPSCs, induced pluripotent stem cells; KGF, keratinocyte growth factor; LuMSCs, lung-resident mesenchymal stem cells; LSC, lung spheroid cell; Mφ, macrophage; MCP1, monocyte chemotactic protein 1; miRNA, micro RNA; MMP9, matrix metalloproteinase-9; NO, nitric oxide; P-10, interferon-γ-induced protein 10; PDGF, platelet-derived growth factor; PEPCs, prominin-1/CD133(+) epithelial progenitor cells; PL-MSCs, placental mesenchymal stem cells; STC1, stanniocalcin-1; TNF-α, tumor necrosis factor-α; TGF-β, transforming growth factor-beta 1; Treg, regulatory T cell; TIMPs, Tissue inhibitors of metalloproteinases; UC-MSCs, umbilical cord mesenchymal stem cells; VEGF, vascular endothelial growth factor. (※ These studies include content on improving the efficacy of stem cell-based therapy, ↑ indicates upregulation or increase. ↓ indicates downregulation or decrease.).

**Table 2:** Clinical trials on cell-based therapy for fibrosing interstitial lung diseases with stem cells.

Clinical Trials									
Year	Patients	Source	Phase		Endpoint	Administration	Outcome	Ref.	
2013	IPF (FVC > 50% and DLCO > 35%)	Human AD-MSC SVF (autologous)	Phase Ib, non-randomized, and non-placebo	n = 14	Adverse events within 12 months	Intratracheal	No cases of serious or clinically meaningful adverse events	J Transl Med. 2013:11:171.	[91]
2014	IPF (moderately severe)	Human PL-MSC (allogeneic) 1 × 10^6^ vs. 2 × 10^6^ cells	Phase Ib and non-placebo	n = 8	Observation: lung function, 6 min walk test, and CT	Intravenous	Lung function, 6 min walk test, and CT scan were unchanged at 6 months	Respirology. 2014;19(7):1013-8.	[93]
2016	IPF (moderate and progressive)	Human AT2	Phase I, non-randomized, and non-placebo	n = 16	Adverse events within 12 months	Intratracheal	No significant adverse events and no deterioration in pulmonary function or respiratory symptoms	Chest. 2016;150(3):533-43.	[89]
2017	IPF (mild to moderate)	Human BM-MSC (allogeneic), 2 × 10^7^, 1 × 10^8^, and 2 × 10^8^ cells	Phase I and non-placebo (AETHER trial)	n = 9	Safety and serious adverse events at 4 weeks	Intravenous	No treatment-emergent serious adverse events	Chest. 2017;151(5):971-981.	[94]
2018	IPF (80% > FVC > 55% and DLco > 35%)	Human AD-MSC SVF (autologous)	Phase Ib, non-randomized, and non-placebo Follow-up of the phase I study (J Transl Med. 2013)	n = 14	Observation: mortality, progression-free survival, lung function, and exercise capacity within 54 months	Intratracheal	Two-year median survival and progression rates are close to epidemiological data.	Clin Respir J. 2018;12(6):2084-2089.	[92]
2019	IPF (mild to moderate)	Human BM-MSC (allogeneic) 2 × 10^7^ vs. 1 × 10^8^ cells	Phase I and non-placebo (data from AETHER trial)	n = 9	Compare CT changes with pulmonary function	Intravenous	Slower progression of lung fibrosis and DLCO in the 1 × 10^8^ group	Eur Rev Med Pharmacol Sci. 2019;23(17):7568-7572.	[95]
2020	IPF (rapid progressive course of severe to moderate)	Human BM-MSC (allogeneic) 2 × 10^8^ cells	Phase I/IIA, randomized, vs. placebo	n = 20	Safety, tolerability, and efficacy	Intravenous	No significant adverse effects; 6 min walk distance (13 wks) ↑, DLCO (26 wks) ↑, and FVC (39 wks) ↑ in BM MSC	Stem Cells Transl Med. 2020;9(1):6-16.	[96]

**Abbreviations:** AD-MSCs, adipose-derived MSCs; AT2, alveolar epithelial type II; BM-MSCs, bone marrow mesenchymal stem cells; CT, computed tomography; DLCO, diffusing capacity of the lung for carbon monoxide; FVC, forced vital capacity; IPF, idiopathic pulmonary fibrosis; PL-MSCs, placental mesenchymal stem cells; SVF, stromal vascular fraction. (↑; indicates improved.)

## Data Availability

Not applicable.
